# Supplementary material for: Puriton attenuates the asthma severity in ovalbumin-induced murine model via balancing Th1/Th2 and inhibiting inflammation
Source: PLoS One. 2025 May 5;20(5):e0322792. doi: 10.1371/journal.pone.0322792 (PMC12052110; doi:10.1371/journal.pone.0322792)
Supplement: Attachment — Submitted filename: New Microsoft Word Document (5).docx [file pone.0322792.s001.docx]

Review:

1. **Justification for Group Division**
   Please provide a detailed justification for the division of 48 mice into five groups. Specify the criteria used for group allocation and describe the experimental rationale for this grouping strategy.
2. **Introduction Quality**
   The introduction is well-structured and effectively written, providing a comprehensive background and rationale for the study.
3. **Rationale for Dividing Experimental Mice for BALF and Histological Analyses**
   Kindly clarify the rationale for dividing experimental mice into two subgroups: one for bronchoalveolar lavage fluid (BALF) analysis (n=4) and the other for histological examination (n=4). Given that these analyses could theoretically be conducted on the same group, explain why splitting the groups was deemed necessary.
4. **Image Visibility in Immunohistochemistry Figures (Figure 2B)**
   Some images in immunohistochemistry figures (2B b and 2B d) are not clearly visible for proper visualization and analysis. Please ensure the quality and resolution of these images are sufficient for accurate interpretation.
5. **Th1/Th2 Cytokine Balance**
   The study demonstrates good connectivity regarding the balance between Th1 and Th2 cytokine release. However, the lack of signaling pathway analysis limits the mechanistic understanding of how Puriton attenuates asthma severity. It is recommended to address this gap without this anti-inflammatory effect of Puriton cannot be justificable.
6. **NF-κB Level Analysis**
   To strengthen the findings, it is suggested that the study should include an analysis of NF-κB levels. This would provide further insights into the underlying molecular mechanisms and support the proposed protective role of Puriton in asthma.
